# The case for launch of an international DNA-based birth cohort study

**Published:** 2011-06

**Authors:** Igor Rudan, Mickey Chopra, Yurii Aulchenko, Abdullah H. Baqui, Zulfiqar A. Bhutta, Karen Edmond, Bernardo L. Horta, Keith P. Klugman, Claudio F. Lanata, Shabir A. Madhi, Harish Nair, Zeshan Qureshi, Craig Rubens, Evropi Theodoratou, Cesar G. Victora, Wei Wang, Martin W. Weber, James F. Wilson, Lina Zgaga, Harry Campbell

**Affiliations:** 1 Centre for Population Health Sciences and Global Health Academy, University of Edinburgh, Scotland, UK; 2 Croatian Centre for Global Health, Faculty of Medicine, University of Split, Croatia; 3 UNICEF Headquarters, New York, USA; 4 Independent Scientist and Consultant, Rotterdam, The Netherlands; 5 Johns Hopkins Bloomberg School of Public Health, Johns Hopkins University, Baltimore, USA; 6 Division of Women and Child Health, the Aga Khan University, Karachi, Pakistan; 7 Infectious Disease Epidemiology Unit, London School of Hygiene and Tropical Medicine, London, United Kingdom; 8 Faculty of Medicine, Federal University of Pelotas, Pelotas, Brazil; 9 The Rollins School of Public Health, Emory University, Atlanta, USA; 10 Instituto de Investigación Nutricional, Lima, Peru; 11 US Navy Medical Research Unit 6, Lima, Peru; 12 Department of Science and Technology/National Research Foundation, University of Witwatersrand & National Institute for Communicable Diseases: Division of National Health Laboratory Services, Johannesburg, South Africa; 13 Neonatology Ward, Edinburgh Royal Infirmary, Edinburgh, Scotland. UK; 14 Center for Childhood Infections and Prematurity Research, Seattle Children’s Met Park West, Seattle, USA; 15 School of Public Health and Family Medicine, Capital Medical University, Beijing, China; 16 Chinese Academy of Sciences, Beijing, China; 17 Department of Child and Adolescent Health and Development, World Health Organization, Geneva, Switzerland

## Abstract

The global health agenda beyond 2015 will inevitably need to broaden its focus from mortality reduction to the social determinants of deaths, growing inequities among children and mothers, and ensuring the sustainability of the progress made against the infectious diseases. New research tools, including technologies that enable high-throughput genetic and ‘-omics’ research, could be deployed for better understanding of the aetiology of maternal and child health problems. The research needed to address those challenges will require conceptually different studies than those used in the past. It should be guided by stringent ethical frameworks related to the emerging collections of biological specimens and other health related information. We will aim to establish an international birth cohort which should assist low- and middle-income countries to use emerging genomic research technologies to address the main problems in maternal and child health, which are still major contributors to the burden of disease globally.

## INTRODUCTION

Progress towards the reduction in child mortality target in Millennium Development Goal (MDG) 4 is being made in many low- and middle-income countries (1). This is mainly as a result of notable efforts of national governments and the international community to improve the prevention and treatment of the main causes of child death and to expand access to health care. The exact causes of the sharp declines in child mortality have not been identified definitively but successful countries have significantly increased coverage of basic public health interventions and increased access to quality health services (1). The ‘advanced market commitment’ programmes, which are heavily supported by The Gates Foundation, Global Alliance for Vaccines and Immunization and national governments of the developed countries, will try to sustain and enhance the development and implementation of vaccination against major pathogens throughout the developing world (2).

Many low- and middle-income countries are now experiencing a markedly different pattern of early mortality, with more than half of child deaths attributable to causes directly related to birth and very early infections and complications – such as pre-term birth complications, birth asphyxia, congenital anomalies, sudden infant death syndrome and accidents (3). At the same time, attention is shifting from not only ensuring survival, but also attaining optimal development. A growing body of evidence suggests that social and environmental influences, especially during pregnancy and in early childhood, can have important long term health and development implications (4). Another important trend that accompanies the epidemiological and demographic transitions is increasing inequities in child health outcomes within countries.

The global child health agenda for 2015 and beyond will inevitably need to broaden its focus from mortality reduction to also addressing the social determinants of deaths, growing inequities among children and mothers, and ensuring the sustainability of the progress made against the infectious diseases. With continued reduction in child mortality, the focus of international efforts will also need to shift from merely averting deaths to promoting better health, development, social functioning and education of children in the poorest countries and reduce the effects of inequity. The research needed to address those challenges will require conceptually different studies than those used in the past to address infectious causes.

## HUMAN GENOME PROJECT AND MEDICAL RESEARCH

The past decade has witnessed remarkable progress in the development of more reliable, replicable and standardized methods that have improved the quality of research in all areas of human health and development. This is true across a wide range of disciplines, from qualitative research and indicators of quality of life or inequity, to cut-ting-edge basic research. In parallel to global child mortality reduction, we have witnessed a particular revolution in biomedical research which was brought about by the progress in genetic technology as a result of the Human Genome Project (5).

Genome-wide association studies and whole-genome sequencing have led to an unprecedented level of discovery with novel insights into human biology and the genetic determinants of many common and rare human diseases. However, common human diseases of late onset – such as cardiovascular diseases, most types of cancer, or type 2 diabetes, which are typical of the industrialized countries, have a complex aetiology, including large numbers of strong environmental, social and behavioural determinants and non-genetic risk factors.

For most traits studied, the contribution of genetic factors associated with those diseases is due to many common genetic variants, each with very small effects (6). More importantly, the research into genetic determinants of common human diseases has been very largely confined to industrialized countries and focused on their health needs, which form a relatively minor part of the global burden of disease (6). Several researchers in the field have expressed concern that genetic research into common diseases of late onset conducted to date has mainly been serving to increase the gap between the health needs of the global poor and the rich (7).

In the past 2 million years of human evolution, diseases of the late onset had little impact on the natural selection processes that shaped the human genome. They occur in a post-reproductive period and are therefore almost invisible to selection. Thus their genetic architecture is likely to be mostly defined by so-called ‘neutral evolution’. Diseases and conditions that had a much greater power to shape human genetic make-up – the problems related to birth, early child survival and maternal mortality – are still present in low-income countries. Those are mainly the problems occurring during labour and persisting infectious causes of mortality of children and young people. They have been shaping the human genome through natural selection. Applying new genetic technologies to study the genetic variants protecting from the major causes of maternal and child deaths would likely yield significant insights into pathogenesis of those diseases and mechanisms of evolved host resistance. Moreover, some of these traits are likely to be subject to balancing and shifting selection, and may reveal larger genetic effects.

Another impact of the human genome project was the development of technologies for fast and relatively cheap sequencing of genomes. Currently, a polymerase chain reaction (PCR) test for a specific infectious agent can be developed in the course of few days through re-sequencing of the genome of an infectious agent, as demonstrated by a recent example during the H1N1 (‘swine’) flu outbreak in 2009.

## LARGE-SCALE BIOMEDICAL SCIENCE

The progress made recently through the application of novel genetic technologies in large population cohorts has also exposed the importance of very large and well-designed studies with adequate power to test study hypotheses. Comparison of small candidate gene studies (which were routinely performed in 1990s and in the beginning of 2000s) with large multi-centre collaborative genome-wide association studies (which became very popular from about 2007) shows that conducting many small studies with insufficient power and without the standardized methodology is an inefficient use of resources and leads to inconsistent and false positive reports in an overwhelming majority of cases. Conversely, large multi-centre studies using rigorous statistical approach lead to reproducible and consistent results.

The need for large scale collaborative research programmes is even more important as research turns to the study of the interaction of genes and environment in causing disease (8). This problem is not limited to genetic research. Information on morbidity and mortality from the diseases of the poor, which form the large majority of global burden of disease and death, is often based on data from relatively few studies conducted in different low-income contexts without standardized methods. They often provide very inconsistent estimates that are not comparable. The recent progress made by genome-wide association studies and, more generally, in the field of genetic epidemiology, has highlighted the importance of ‘biobanks’. These are large collections of biological material and extensive associated epidemiological data from large population cohorts assembled in a standardized way. They are designed and developed with close attention to quality assurance, so that emerging technologies can be applied to generate high quality reproducible data on very large numbers of well characterised individuals. One of the main reasons why the developing world is not enjoying potential benefits from the application of novel epidemiological and genetic research technologies is because there are very few biobanks and large population cohort studies in low- and middle-income countries available for systematic study (see Campbell et al. (9) and McKinnon et al. (10) in this issue). Such cohorts are becoming a pre-requisite for reliable research into biological, environmental, genetic, behavioural and social determinants of diseases relevant to their population.

## THE CASE FOR LAUNCH OF AN INTERNATIONAL DNA-BASED BIRTH COHORT STUDY

The development of a very large dataset (‘biobank’) that would include or contain directly comparable data on epidemiological, biological and social factors and samples representative of a large number of human populations would allow researchers to study the determinants of diseases which have made major contributions to the global burden of disease, thus reducing the large inequity in research effort on different causes of morbidity and mortality globally. An international biobank would allow researchers in low- and middle-income countries to gain access to emerging research technologies and use them to study diseases that affect their populations. It would also serve to build and enhance the research capacity in developing countries, to create a large collaborating network of interested researchers globally, and to enable comparisons between their results using the same methodology. The results of biomedical research that would be generated from such a biobank would make a substantial contribution to improving the available information on the morbidity, mortality and the main risk factors for the diseases relevant to the developing world and provide the opportunity to study interactions between genes and a wide range of levels of environmental exposures in causing diseases in different contexts (8). The longitudinal nature of the study could provide valuable information on long-term (life-course) effects of biological, environmental, genetic, social and other factors and their interplay in the modern age.

The development of a global birth cohort would hold out the promise of understanding genetic, environmental and social determinants of health, development and survival of pre-school children. It would also enhance our understanding of the interactions of context-specific social, economic and environmental factors and the human genetic makeup. Ultimately, what will start as a cohort of newborns and children will eventually, over the course of the 21st century, develop into a longitudinal cohort study of adolescents, and then adults with multiple data points through a time series. This will allow testing many current hypotheses on the causal role of different major risk factors – social, environmental, behavioural and genetic – in human disease and provide answers relevant to all human populations, rather than being limited to high-income countries. It would create a reliable information base that could assist in understanding the burden of health problems in mothers and children globally. There would also be a role for this resource to help understand the genetic and other factors that make children prone to poor response or adverse effects of medicines or vaccines. The conduct of this coordinated study in a consistent way across many sites from low- and middle-income countries, starting with several ‘core’ sites and expanding through addition of other sites interested in following the same methodological approach, and including up to 1 million newborns, would maximise the power to address many high priority research and health care system questions. Results would also be generalisable globally, rather than confined to specific ethnic, social or economic groups. Through parallel recruitment and involvement of mothers, it would serve to integrate research into interlinked maternal and child health problems.

**Figure:**
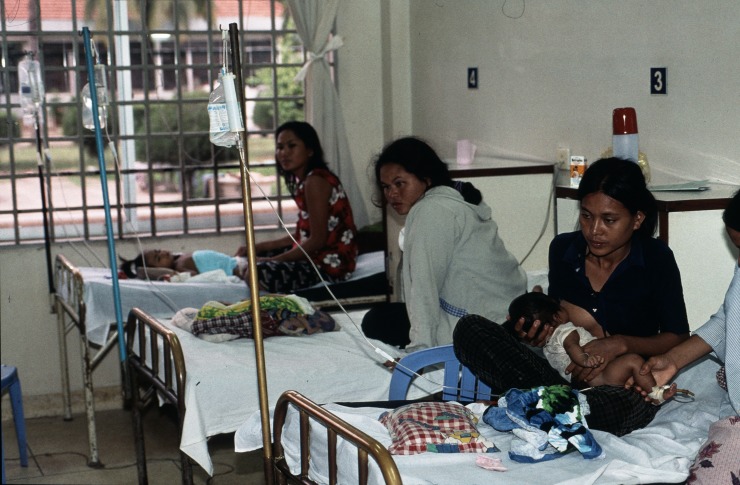
Photo: Courtesy of Dr Martin Willi Weber, personal collection

Building up an all-inclusive international biobank based on best research practice would, over time, develop into a resource that could serve the research needs of many diverse groups of researchers from developed and developing countries alike. The principle that would underlie this cohort is adherence to the highest ethical standards, which have now been developed through the setting up of large biobanks in developed countries, and also the principles of open access to anonymised data (consistent with adherence to ethical principles and local approvals) to interested researchers with legitimate research ideas. This is in line with the emerging ‘open science’ framework, which enabled open discussion and reanalysis of existing data. Stringent adherence to the highest ethical standards needs to be emphasized throughout each study as one of the goals of the larger project. The development of an international biobank would involve the human and technological capacity of developing countries to work on this project and to assist in building their research capacity and competitiveness.

In many countries there will be a strong sensitivity towards allowing foreign institutions to access genetic material. Most poor countries will rightly fear that any commercial interests from this type of research will only benefit rich countries. Some of them already have laws in place which restrict sending any biological materials outside their borders, particularly those that may have commercial implications. This is why planning an international birth cohort based on DNA will require developing of local capacity. Local researchers will need to be trained to obtain biological materials in the same way across many field sites, store them properly, and generate data locally, while the analysis of the data could then be standardized and centralized. Preserving commercial interests from any patents and sharing them fairly with all the participants from low and middle income countries will represent an important challenge.

## WHY IS THE TIME RIGHT?

We need a vision beyond the MDG4 timeframe of 2015. It is time to move beyond focusing on simply averting child deaths and to start planning for this resource. We now have standard operating procedures developed specifically for setting up biobanks and validated genetic technologies and analytical methods for replicable and reliable genetic analyses (11,12). We also have standard ethics principles that are applicable to biobanks and large experience in setting up biobanks in high-income countries (13). Greatly improved communication globally, through internet and mobile phones, and cheaper international travel, has enabled a new kind of research. It is based on massive collaborations of scientists, big projects and large sample sizes, and it generates more reliable results. We propose that globalization of the lifestyle, industry, and many other segments of human activity should be followed by globalization of research into human health and development. There is increasingly a technological capacity in developing countries that could support this kind of vision. For example, several large international organizations, multilateral agencies and even donor foundations have both the legitimacy and organisational infrastructure to provide parallel access to many field sites in low-income countries. They also represent a well-known ‘brands’ in the international community that could ensure wide participation and commitment from all those taking part in this study. Some organizations and agencies maintain programmes of regular and repeated contacts with a high proportion of children in low resource settings, which could provide a highly cost-efficient framework for recruitment and longitudinal follow up throughout early childhood.

In addition, the incredibly fast progress in development of supporting genetic research technologies, which led to many recent genetic breakthroughs through genome-wide association studies, is beginning to make this vision increasingly realistic. In fact, technological advances are now the main driver of the research progress. It can already be assumed that the appropriate research technologies will become affordable in several years to perform large-scale whole-genome sequencing projects. The costs of genotyping and sequencing of the human genome have been falling rapidly since the year 2000 – from about US$3 billion (€2.1 billion), which was spent to sequence the first human genome, to only about US$ 4000 (€ 2800) in early 2011 (14,15). The time required to sequence the entire human genome has also fallen from 11 years to only a few hours (14,15).

We need to anticipate the possibility of affordable mass-sequencing several years from now. With this vision, the remaining time would be well spent developing and assembling the datasets from many countries and designing studies which will be the most informative, assuming the availability of genomic information (16).

## WHAT SHOULD BE THE STUDY DESIGN OF GENERATION 2015?

The study sample should be large and represent many of the world’s populations. In each country, a local academic expert in paediatrics/neonatology or obstetrics would be identified and would be responsible for obtaining ethics approval from the nationally relevant body to conduct the study. This person should also be a key / committed member of the study team and should own and drive the process at the country level.

The study would typically involve one large urban teaching hospital and 3–4 health facilities in less developed and rural regions. In each country, a proportional number of newborns and mothers would be recruited to achieve a global sample size of up to 1 million newborns and as many of their mothers and fathers. A sample size of this magnitude is required to ensure sufficient number of cases with different social, economic, behavioural, health and development outcomes in different contexts for an adequate, globally representative study. Each pregnant woman would be informed about the goals of the study and she would be asked to give informed consent for herself and the newborn. In some countries, the signature of the father will also be required. At birth, a baseline questionnaire with the basic information would be filled out for each mother and child, and a blood sample would be obtained from each participant. Any complications during birth would be recorded, as well as the basic anthropometric, clinical and psychomotor assessment of the child.

## RESEARCH OUTCOMES AND BENEFITS OF GENERATION 2015

Likely short term research outcomes of Generation 2015 for children would be:

• Description of the morbidity and mortality associated with major child diseases, preterm birth and pregnancy complications;

• Description of normal and abnormal growth and development; and of health and disease from foetal life until early adulthood;

• Identification of biological, environmental, genetic, and social risk factors and their interactions for major child diseases and pregnancy complications in different contexts (including those associated with low birth weight, preterm birth and congenital anomalies; eclampsia, antepartum haemorrhage, placental abruption, thromboembolism and postpartum sepsis);

• Identification of biological, environmental, genetic, and social determinants of host resistance against the major neonatal and childhood infections (such as neonatal sepsis, pneumonia, diarrhoea) and determinants of immune system development;

• Identification of biological, environmental, genetic, and social determinants of malnutrition and stunting (including micronutrient deficiencies);

• Identification of biological, environmental, genetic, and social determinants of child development (motor, behavioural, cognitive and psychomotor development);

• Understanding of genetic and environmental determinants of host-pathogen interactions, carriage of microorganisms, immune response to vaccination, and antibiotic resistance.

• Linking the whole genome studies to similar studies that are starting on global pathogens (17).

For mothers, short term research outcome could include:

• Description of the mortality and morbidity associated with the major maternal diseases and pregnancy complications;

• Identification of biological, environmental, genetic, and social risk factors for these conditions and their complications (including eclampsia, placenta previa, lactation duration, postpartum haemorrhage, twinning, obstructed delivery, and puerperal sepsis) in different contexts.

Research outcome for participating countries could be:

• Training of local staff and development of local research capacity, establishing local biobanks and international competitiveness in biobanking;

• Possibility to participate in research at a high international level through large collaborations and to compare its capacity and progress in maternal and child health research with other countries.

Finally, likely indirect research opportunities afforded by Generation 2015 would be:

• Identification of genes, gene-environment and gene-social interactions;

• Identification of biomarkers for birth related and early childhood disease outcomes or adverse events;

• Establishing global standards for the frequency of gene variants in different populations;

• Defining the content of a genotyping array containing the major disease associated variants across all global population groups;

• Studying the effects of urbanization on genetic structure of populations and impact of admixture on disease traits;

• Studying of the migrations of historical human populations;

• Looking for ‘signatures’ of natural selection in the genome.

## WHAT IS THE PROPOSED STRATEGY OF DEVELOPMENT OF GENERATION 2015?

In the first phase, several ‘core sites’ would be chosen in low- and middle-income countries, in which the approach would be piloted. The sites would fall in one of two categories: 1) they would be set up in each one of the BRICS countries – Brazil, Russia, India, China and South Africa – ie, the five very large and rapidly developing economies. Those five countries are front-runners among the developing nations and their economic potential and research capacity are both on a remarkable increase. These countries should have most interest in, and ability to, harbour study sites in which DNA-based birth cohorts could be developed, as a logical next step necessary to acquire competitiveness in genomic research at an international level; 2) they would be set up in several rare sites in low-income countries in which high-quality research was being conducted for many years, and study populations are used to research of effectiveness of different interventions. These study sites were set up by driven individuals from low-income countries, frequently through bilateral collaboration with a western institution. Because of randomized controlled trials that are being conducted at such sites, the studies have availability of ‘cold chains’ required for vaccine delivery, appropriate ethics approvals, and a motivated team of researchers and study populations. They represent rare and relatively unique sites where a proper DNA-based biobank could be set up.

The pilot studies in those several selected sites would improve our knowledge and understanding of the challenges with developing biobanks in low- and middle-income countries. We would aim to develop standardized study protocols based on those early experiences, and then invite groups from many more low- and middle-income countries to join this Generation 2015 with their data collections assembled in a standardized way. Through this ‘snowballing’ development, we would hope to achieve sufficiently large numbers to develop a true international DNA-based birth cohort, which would allow studying of genetic determinants of health and disease in children across the world, but also try to document the effects of inequity and socioeconomic differences on children’s biology and development potential.
